# Comparison of Machine Learning Methods and Conventional Logistic Regressions for Predicting Gestational Diabetes Using Routine Clinical Data: A Retrospective Cohort Study

**DOI:** 10.1155/2020/4168340

**Published:** 2020-06-12

**Authors:** Yunzhen Ye, Yu Xiong, Qiongjie Zhou, Jiangnan Wu, Xiaotian Li, Xirong Xiao

**Affiliations:** ^1^Obstetrics and Gynecology Hospital, Fudan University, Shanghai, China; ^2^The Shanghai Key Laboratory of Female Reproductive Endocrine-Related Diseases, Shanghai, China; ^3^The Shanghai Key Laboratory of Birth Defects, Shanghai, China; ^4^Institute of Biochemical Science, Fudan University, Shanghai, China

## Abstract

**Background:**

Gestational diabetes mellitus (GDM) contributes to adverse pregnancy and birth outcomes. In recent decades, extensive research has been devoted to the early prediction of GDM by various methods. Machine learning methods are flexible prediction algorithms with potential advantages over conventional regression.

**Objective:**

The purpose of this study was to use machine learning methods to predict GDM and compare their performance with that of logistic regressions.

**Methods:**

We performed a retrospective, observational study including women who attended their routine first hospital visits during early pregnancy and had Down's syndrome screening at 16-20 gestational weeks in a tertiary maternity hospital in China from 2013.1.1 to 2017.12.31. A total of 22,242 singleton pregnancies were included, and 3182 (14.31%) women developed GDM. Candidate predictors included maternal demographic characteristics and medical history (maternal factors) and laboratory values at early pregnancy. The models were derived from the first 70% of the data and then validated with the next 30%. Variables were trained in different machine learning models and traditional logistic regression models. Eight common machine learning methods (GDBT, AdaBoost, LGB, Logistic, Vote, XGB, Decision Tree, and Random Forest) and two common regressions (stepwise logistic regression and logistic regression with RCS) were implemented to predict the occurrence of GDM. Models were compared on discrimination and calibration metrics.

**Results:**

In the validation dataset, the machine learning and logistic regression models performed moderately (AUC 0.59-0.74). Overall, the GBDT model performed best (AUC 0.74, 95% CI 0.71-0.76) among the machine learning methods, with negligible differences between them. Fasting blood glucose, HbA1c, triglycerides, and BMI strongly contributed to GDM. A cutoff point for the predictive value at 0.3 in the GBDT model had a negative predictive value of 74.1% (95% CI 69.5%-78.2%) and a sensitivity of 90% (95% CI 88.0%-91.7%), and the cutoff point at 0.7 had a positive predictive value of 93.2% (95% CI 88.2%-96.1%) and a specificity of 99% (95% CI 98.2%-99.4%).

**Conclusion:**

In this study, we found that several machine learning methods did not outperform logistic regression in predicting GDM. We developed a model with cutoff points for risk stratification of GDM.

## 1. Background

Gestational diabetes mellitus (GDM) is a disease in which carbohydrate intolerance develops during pregnancy [1]. GDM affects approximately 14.8% of pregnant mothers in China [2], and the prevalence of GDM is increasing worldwide [3]. Women with GDM undergo metabolic disruption and placental dysfunction [4], increasing the risks for preeclampsia and cesarean delivery [5]. Hyperglycemia and placental dysfunction adversely affect fetal development, increasing the risks of birth trauma, macrosomia, preterm birth, and shoulder dystocia [6, 7]. The mother with GDM and her offspring are both more likely to develop obesity, type 2 diabetes mellitus, and cardiovascular disease than those without GDM [8, 9].

Early diagnosis and intervention should decrease the incidence of GDM and lower adverse pregnancy outcomes [10, 11]. However, based on most guidelines, most GDM cases are diagnosed between 24 and 28 gestational weeks by an oral glucose tolerance test (OGTT) [12, 13], which may miss the optimal window for intervention, as fetal and placental development have already occurred by this point [14]. Universal diagnosis by OGTT at early pregnancy has been suggested [15], but this is costly and inefficient as in most cases, GDM manifests during mid-to-late pregnancy [16]. Overall, early prediction is needed and may be valuable. Developing a simple method using the existing clinical data at early pregnancy to quantify a woman's risk of developing GDM would help to identify high-risk mothers in need of early diagnosis, monitoring, and therapy and serve to obviate universal OGTTs for low-risk women [10].

Recent prediction models for GDM have been developed using conventional regression analyses [17–19]. However, machine learning, a data analysis technique that develops algorithms to predict outcomes by “learning” from data, is increasingly emphasized as a competitive alternative to regression analysis. Moreover, machine learning has the potential to outperform conventional regression, possibly by its ability to capture nonlinearities and complex interactions among multiple predictive variables [20]. Despite this, only four studies [21–24] have used machine learning algorithms to predict GDM, and none of them compared their performance with that of logistic regressions.

In this study, we aimed to use machine learning methods to develop a model incorporating data on maternal characteristics and biochemical tests to predict the presence of GDM and to compare their performance with that of traditional logistic regression models. It is hypothesized that machine learning algorithms outperform traditional logistic regression models in terms of discrimination and calibration.

## 2. Materials and Methods

### 2.1. Study Population and Data Source

#### 2.1.1. Study Setting

A single-center, retrospective cohort study was conducted to derive and validate a model with cutoff points for the prediction of GDM. Eligible subjects were women with singleton pregnancies who had records of serum samples collected before 24 gestational weeks for prenatal biochemical examination and Down's syndrome screening and who later delivered at the Obstetrics and Gynecology Hospital of Fudan University from 2013 to 2017. All women with multiple pregnancies or previous diabetes were excluded. Informed consent was obtained from all the participants, and the study protocol was approved by the Ethics Committee of Gynecology and Obstetrics Hospital of Fudan University.

#### 2.1.2. Predictive Variables

Predictor variables included medical history, clinical assessments, ultrasonic screening data, biochemical data of liver/renal/coagulation function at the prenatal visit, and data from Down's syndrome screening. In total, 104 variables were assessed. Briefly, medical history included a history about diabetes and previous pregnancy and the woman's family history. Clinical assessment included maternal age, educational status, smoking, body mass index (BMI), and parity. Biochemical tests at the first prenatal visit were performed after fasting for at least 8 h. Down's syndrome screening was performed between 16 and 20 gestational weeks, and ultrasound screening was performed between 24 and 28 gestational weeks.

#### 2.1.3. Outcomes

The primary outcome was GDM, which was diagnosed according to IADPSG criteria 2010 [12]. Briefly, GDM was defined according to the 75 g OGTT (0-1-2 hours: 10.1-8.5-5.1 mmol/L) from 24 to 28 gestational weeks, and the diagnosis of GDM was established if any single glucose concentration met or exceeded a fasting value of 5.0 mmol/L, a 1 h value of 10 mmol/L, or a 2 h value of 8.5 mmol/L.

The secondary outcome was adverse pregnancy outcomes, including cesarean delivery for any reason, preeclampsia, macrosomia (birth weight ≥ 4000 g), IUGR (intrauterine growth restriction), preterm birth (≤34 gestational weeks), neonatal asphyxia (Apgar score ≤ 3), and perinatal death. These outcomes were evaluated because of their reported associations with GDM [25].

### 2.2. Study Objectives and Strategies

The primary objective was to compare the performance of various machine learning models and conventional logistic regression models by discrimination and calibration. The second objective was to estimate an optimal model with one point (at or above) to predict the presence of GDM and with another point (at or below) to predict the absence of GDM.

### 2.3. Model Development

#### 2.3.1. Overview

**Figure 1:**
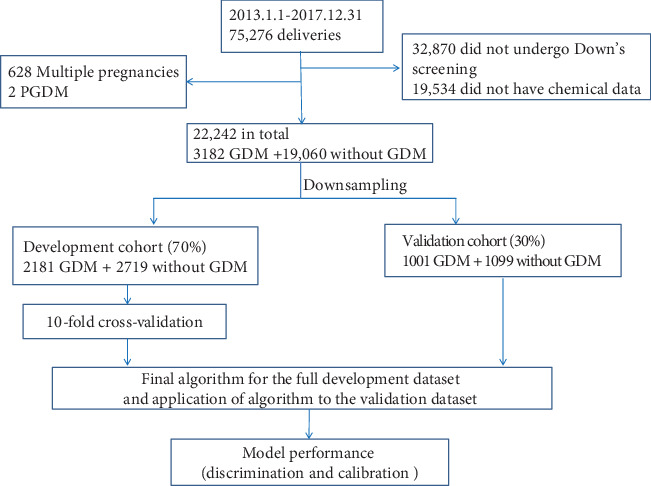
Study profile and analysis pipeline.

We conducted the study to derive and validate a model for the prediction of GDM by means of a two-phase approach (development and validation). The dataset of the GDM group was randomly split into the development (70%) and validation (30%) cohorts. The dataset of women without GDM was randomly downsampled at a 1 : 1 ratio into the GDM group to obtain balanced data. Thus, in the development phase, we used data from 4900 participants (2181 with GDM and 2719 without GDM) to derive a model and its cutoff to predict the presence of GDM, and this cutoff was validated with the use of data from 2100 additional participants (1001 with GDM and 1099 without GDM) (Figure 1).

#### 2.3.2. Data Processing

First, we extracted information for each specific index from the records and converted their descriptions to numerical variables. Some numerical variables were processed hierarchically and transformed into categorical variables; for example, patient biochemical tests were classified into categories, namely, normal and abnormal test results. Additionally, variables pertaining to patient characteristics were converted to numerical variables.

Second, missing data were calculated, and they were eliminated if more than 40% of the data were missing for one participant; otherwise, the missing data were filled in by means of the mean filling method.

Third, the Pearson correlation coefficient was used to calculate the correlation coefficient between two features, aiming to judge the multicollinearity. A feature was eliminated if the absolute value of the correlation coefficient was higher than 0.75, as this indicated a strong collinearity.

Data standardization is a basic work of data mining. Different features often have different dimensions and dimension units, which will affect the results of the data analysis. To eliminate the dimensional impact among features, data standardization is needed to solve the comparability between data features. After the original data are standardized, each index is on the same order of magnitude, which is suitable for comprehensive evaluation. In the present study, the method of deviation standardization was used to normalize the continuous characteristic variable to 0-1.

#### 2.3.3. Development Phase

Eight machine learning algorithms and two conventional logistic regressions were used to develop predictive models. All model tuning using ten-fold cross-validation was performed in the development dataset. Multiple methods were used to derive the model from the same set of data. Results for recall, precision, and *F*-measure were obtained.

#### 2.3.4. Conventional Logistic Regression Models

Two conventional logistic regression models were compared in this study. The first model was fit with each variable entered linearly. The second model used restricted cubic splines with three knots to allow for nonlinearity. In both models, maternal age, BMI, and previous GDM were always included in the model, regardless of statistical significance, because they have been reported to be associated with GDM. Other predicted variables were retained in the model if they were statistically significant between the GDM and control groups (*P* < 0.05).

#### 2.3.5. Machine Learning Models

*(1) GBDT*. GBDT is an integrated learning model based on a decision tree that adopts the additive model method. In the iterative training process, the model generates a weak classifier based on the residual of the last iteration and achieves the purpose of data classification by constantly reducing the residual generated in the training process.

*(2) AdaBoost*. AdaBoost is also an integrated learning algorithm. In the iterative process, the algorithm generates a new learner on the training set to predict all samples and evaluate the importance of each sample. The more difficult it is to distinguish samples, the higher the given weight will be in the iterative process. The whole iterative process is terminated when the error rate is small enough or a certain number of iterations is reached.

*(3) LGB*. LGB uses a decision tree based on a learning algorithm. LGB uses a histogram algorithm to divide the continuous floating-point features into *k* discrete values and constructs a histogram with a width of *K*. Then, the training data are traversed, and the cumulative statistics of each discrete value in the histogram are calculated. In feature selection, only the discrete value of the histogram is needed to traverse to find the optimal segmentation point.

*(4) Vote*. In the present study, Vote was used to synthesize the results of other algorithms in the present study; that is, when the samples were predicted and judged, other algorithms were used to vote, and the category with the most votes was the output result of the vote algorithm.

*(5) XGB*. XGB establishes *K* regression trees to make the predicted value of the tree group close to the real value as much as possible and has the ability to generalize as much as possible. The objective function of XGB requires the prediction error to be as small as possible, the number of leaf nodes to be as small as possible, and the number of nodes to be as low as possible.

*(6) Random Forest*. Random forest is a nonlinear tree-based integrated learning model. Random forest establishes a forest in a random way. The forest is composed of many decision trees, and there is no correlation between each decision tree. After the random forest model is obtained, each decision tree in the random forest is judged when the new sample enters. For the classification problem, the voting method is used, and the maximum number of votes is the final model output.

*(7) Decision Tree*. The decision tree is a basic classification method. The decision tree consists of nodes and directed edges. A decision tree contains a root node, an internal node, and a leaf node, in which the internal node represents a feature and the leaf node represents a class. First, the feature is filtered according to the information gain of the feature. Then, each node is divided into subnodes according to the feature value. The root node contains the sample set. The path from the root node to each leaf node corresponds to a decision sequence.

*(8) ML Logistic*. Logistic regression, also known as logarithmic probability regression, is a classification model and suitable for the fitting of numerical binary output data. After the input data are linearly weighted, a sigmoid function is used to process the input data to obtain the output probability result, and then, the probability result is transformed into binary output by a symbol function. The parameters of the input model are obtained by maximum likelihood estimation, which distinguishes it from conventional logistic regressions.

#### 2.3.6. Validation Phase

Predictive probabilities were calculated for each model in the validation dataset from each development model in two ways. First, model discrimination was assessed by the area under the receiver operating characteristic (AUC) curve, where a value of 1.0 represents perfect discrimination and 0.5 represents no discrimination. Second, model calibration was assessed by the mean square errors.

To estimate the relative contribution of the variables in the best performing machine learning methods and logistic regressions, the AUC-based permutation importance measure for the best performing machine learning method and the Wald *χ*^2^ statistics minus the degrees-of-freedom (*χ*^2^ − *df*) for the best performing logistic regression were computed. The effects of the most accurate predictor variables across different values and predicted risk were evaluated for the most accurate model using partial dependence plots. The distribution of predictive value versus its percentage in the whole population or occurrence of GDM is shown. Distributions of predictive values in either the development or validation dataset are also shown.

The negative predictive value, positive predictive value, sensitivity, specificity, positive likelihood ratio, and negative likelihood ratio were obtained. Cutoff points were selected from the development cohort to achieve a negative predictive value higher than 85% or a positive predictive value higher than 85%, which were further analyzed in the validation cohort. All analyses were performed by R version 3.6.1 or python3.6.5. A single-tailed *P* value < 0.05 was regarded as statistically significant.

## 3. Results

### 3.1. Baseline Characteristics

A total of 22,872 mothers were enrolled (Figure 1) from 2013 to 2017. The analysis included 22,242 participants who could be further evaluated (Figure 1). The incidence of GDM was 14.31% in the total cohort (Figure 1). The proportions of overweight, older age, history of prior GDM, and family history of diabetes among mothers who developed GDM were higher than those among mothers who did not develop GDM (Table 1). There were no statistically significant differences in the rates of nulliparity, prior macrosomia, or preterm delivery (Table 1).

### 3.2. Model Comparisons

**Figure 2:**
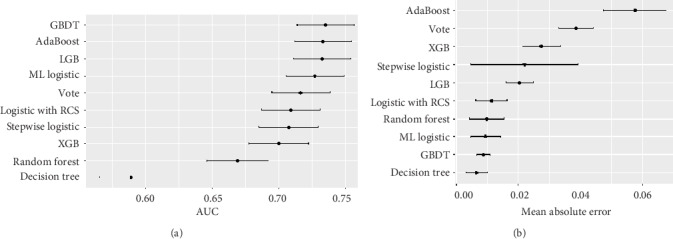
Results of discrimination and calibration metrics of machine learning and logistic regressions in the validation cohort. The AUC (a) and mean absolute error (b) are presented in each model as mean and 95% confidence intervals.

Figure 2 represents the discrimination and calibration performance of machine learning and logistic regression models. In terms of discrimination, the AUCs of the best performing machine learning model (GDBT) and the best performing logistic regression model (logistic with RCS) were similar (73.51%, 95% CI 71.36%-75.65% vs. 70.9%, 95% CI 68.68%-73.12%). The decision tree model was the least discriminative (59.96%, 95% CI 57.53%-61.4%). In terms of calibration performance, indexed by the mean square error, the decision tree (65%, 95% CI 0.30%-1.0%) was the best calibrated model, followed by the GBDT model (0.88%, 95% CI 0.65%-1.1%).

**Figure 3:**
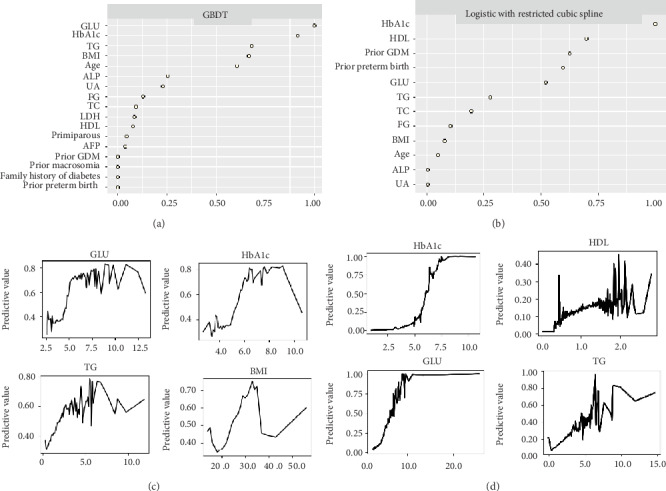
Contribution of the predictor variables in GBDT and the logistic regression model. (a) Importance of the predictor variables in the GBDT model in the validation cohort. (b) Importance of the predictor variables in the logistic model with restricted cubic spline in the validation cohort. (c) Partial plot of the effects of fasting blood glucose (GLU, mmol/L), glycosylated hemoglobin (HbA1c, %), triglyceride (TG, mmol/L), and maternal BMI (kg/m^2^) on the risk of GDM across different values in the GBDT model. (d) Partial plot of the effect of glycosylated hemoglobin (HbA1c, %), high-density lipoprotein (HDL, mmol/L), fasting blood glucose (GLU, mmol/L), and triglyceride (TG, mmol/L) on the risk of GDM across different values in the logistic model with restricted cubic spline.

Figure 3 shows the top ten predictor variables in the GBDT (Figure 3(a)) and logistic regression models (Figure 3(b)). In both models, fasting blood glucose, HbA1c, triglycerides, and maternal BMI were among the most important predictors. However, the top four predictor variables were not completely similar. Fasting blood glucose, HbA1c, triglycerides, and maternal BMI were the most important predictor variables in GBDT (Figure 3(a)), while HbA1c and high-density lipoprotein were the most important predictor variables in the logistic regression (Figure 3(b)). In both models, the risks for GDM increased with increasing levels of the predictors (Figures 3(c) and 3(d)).

### 3.3. Optimal Model Analysis

**Figure 4:**
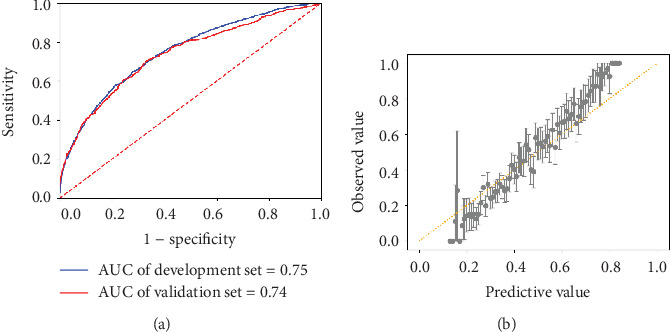
Predictive performance of the GBDT model. (a) AUCs of the development and validation cohorts. (b) The graph presents the calibration curve of the GBDT model by showing the relationships between observed and predicted GDM using the GBDT model. Data are presented as mean and 95% CIs.

Further analyses were performed in the GBDT model. The AUCs of GBDT in both the development and validation cohorts are shown in Figure 4 (75%, 95% CI 73.42%-76.22% vs. 74%, 95% CI 71.36%-75.65%). Model calibration was shown by comparing the observed and model-predicted risk of GDM in the general cohort (Figure 4(a)). At or below the cutoff point of 0.3, the observed occurrence was slightly lower than that predicted by the model, while at or above the cutoff point of 0.7, the observed occurrence was slightly higher than the predicted value; however, overall, the predicted risk fits the observed occurrence well (Figure 4(b)).

**Figure 5:**
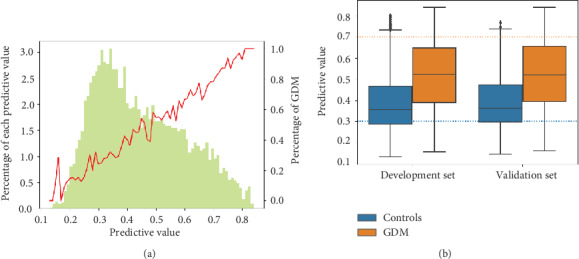
Predictive values for participants with and without GDM in the cohorts. (a) Distribution of predictive value and its relationship with the observed GDM in the combined cohort (development and validation cohort). (b) Distribution of predictive values in the development and validation cohort. Data are presented as median and interquartile ranges.

The distribution of the predictive values in the combined cohorts is presented in Figure 5, which shows skewness, and the top distribution was at a cutoff of 0.3. The median of the predictive value was 0.42. The occurrence of GDM increased linearly with increasing predictive value (Figure 5(a)). A total of 569 of the participants had predictive values at or above 0.7, and 89.3% of them developed GDM, accounting for 16% of the cases of GDM (Figure 5(a)).

In the development cohort, the median predictive value was elevated among participants who developed GDM compared with those who did not develop GDM (52.38% vs. 35.48%) (Figure 5(b)). The selected cutoff points derived from the development cohort were 0.3 (rule out) and 0.7 (rule in). At or below the cutoff point of 0.3, the negative predictive value for GDM was 82.4% (95% CI 79.9%-84.7%) with a sensitivity of 8% (95% CI 6.9%-9.2%) (Table 2), while at or above the cutoff point of 0.7, the positive predictive value for GDM was 86.6% (95% CI 82.9%-89.6%) with a sensitivity of 16% (95% CI 14.5%-17.6%) (Table 2).

In the validation cohort, the median predictive values of the participants who developed GDM and those who did not develop GDM were 0.52 and 0.37, respectively (Figure 5(b)). At the cutoff point of 0.3, the negative predictive value was 74.1% (95% CI 69.5%-78.2%) in the validation cohort, and the corresponding sensitivity was 90% (95% CI 88.0%-91.7%) (Table 2). Additionally, at the cutoff point of 0.7, the positive predictive value was 93.2% (95% CI 88.2%-96.1%), with a high specificity of 99% (95% CI 98.2%-99.4%) and a high positive likelihood ratio of 15 (95% CI 14.38-15.61) (Table 2).

### 3.4. Pregnant Adverse Outcomes

In terms of discrimination, the AUCs of GDBT for adverse pregnancy outcomes was 0.68 (95% CI 0.67-0.70) in the development cohort and 0.63 (95% CI 0.61-0.65) in the validation cohort (supplementary file, Figure S1A). At or below the cutoff point of 0.4, the prediction for adverse pregnancy outcomes was slightly lower than the observed predictive values, while at or above the cutoff point of 0.6, the predictive value was slightly higher than the observed value (supplementary file, Figure S1B). The distribution of predictive values in the total cohort was near normal, and the occurrence of adverse pregnancy outcomes increased with increasing predictive value (supplementary file, Figure S2). The median predictive value for adverse pregnancy outcomes seemed higher than in participants without adverse pregnancy outcomes in both the development and validation cohorts, although there was no statistical significance (supplementary file, Figure S2).

In the validation cohort, at the cutoff point of 0.3, the sensitivity was 99% (95% CI 98.3%-99.5%), while the corresponding negative predictive value was 52.4% (95% 32.4%-71.7%) (Table 3). Additionally, at the cutoff point of 0.7, the specificity for GDM was 99% (95% CI 98.1%-99.4%), while the positive predictive value was 79.2% (95% CI 66.5%-88.0%) and the positive likelihood ratio was 4 (95% CI 3.33-4.67) (Table 3).

## 4. Discussion

### 4.1. Main Findings and Significance

We found no evidence to support the hypothesis that GDM prediction models based on machine learning lead to better performance than models based on logistic regression. In the GBDT model, the best performing machine learning model, we identified cutoff points of 0.3 and 0.7 for the predictive value as potential predictors of the absence and presence of GDM, respectively. Our study provides value for risk assessment of GDM.

### 4.2. Comparisons and Interpretations

Previous studies showed inconsistent results regarding the performance of machine learning algorithms compared with regression models [26–28]. In the current analysis, machine learning was not superior to logistic regression. There may be several reasons for this inconsistency. First, logistic regressions are suitable for simple data with linear relationships between variables and outcomes. Failure in controlling variables entering models according to prior knowledge and in addressing the collinearity between variables will result in poor performance. In the present study, work was done in these respects. The Pearson correlation coefficient was used to calculate the correlation between variables, and the method for entering variables into the model was also controlled clinically and statistically. Second, numerous types of machine learning models and logistic regressions may fit and perform differently in different datasets. Eight common machine learning algorithms in the present study were analyzed and compared, and GBDT was identified as the best model with higher discrimination and calibration than the others. Variables in the GBDT model were shown to be nonlinear, underscoring the advantage of identifying nonlinear variables.

In the present study, even if the overall performance of the machine learning model was similar to logistic regressions, the former was employed for further analysis because of the flexibility in managing nonlinear relationships between variables. Most models showed moderate discrimination, with AUCs mostly above 0.70, which is consistent with previous findings [21, 22, 24, 29]. Previous studies have investigated models derived from electronic health records for the predictions of GDM in machine learning models or conventional logistic regressions, but these studies included fewer participants than ours, did not compare the two methods, involved limited variables, or did not comprehensively estimate the performance of discriminations and calibrations [18, 21, 22, 24, 29]. The current studies extend these previous studies by validating predictive ratio cutoff points of 0.3 and 0.7 originating from general maternal characteristics and biochemical data by comparing the performance of machine learning and traditional logistic regression models for the prediction of GDM. Furthermore, our findings underscore the roles of glucose and lipid metabolism in the development of GDM [4, 30]. In both machine learning and logistic regressions, we identified blood glucose, lipids, BMI, and maternal age as important contributors to GDM.

### 4.3. Significance

In predicting GDM, at or above the cutoff point of 0.7, the observed positive predictive value was 93.2%, and the corresponding sensitivity was 99%, representing values in predicting the presence of GDM and identifying high-risk GDM women. Following the identification of high-risk women, proper management, including early diagnosis by OGTT, lifestyle changes, and exercise, may be beneficial in controlling maternal and neonatal complications [31], although additional data are needed.

A predictive value of 0.7 or higher also had value in predicting the presence of fetal adverse outcomes, including macrosomia, preterm delivery, and low Apgar scores, as well as cesarean delivery and preeclampsia, with a positive predictive value of 79.2% and a specificity of 99%. We did not further evaluate the predictive performance separately for maternal and fetal outcomes, as data on corresponding outcomes were few.

The sensitivity values of GDM and adverse pregnancy outcomes at the cutoff point of 0.3 were 90% and 99%, respectively, indicating that most of the GDM and adverse pregnancy outcomes occurred at or above the predictive value of 0.3. Thus, women with a predictive value below 0.3 may potentially avoid further OGTT, although this needs to be further demonstrated in prospective cohorts.

Overall, the prediction model in the present study provides values in screening strategy based on risk assessment for detecting GDM. Pregnant women may benefit from the strategy of allowing for either the elimination of the need for further screening in low-risk women and the initiation of early prevention and treatment measures in high-risk women.

### 4.4. Strengths and Limitations

The strengths of our study are the large pregnancy cohort with data on obstetrical history, clinical assessments, and biochemical variables in early pregnancy. This is the first prediction model with cutoff points for GDM with easy-use clinical data derived from machine learning algorithms and conventional regressions.

There were some limitations in our study. First, the data come from only one institution and lack external validation. Potential bias may occur in the present study. Further prospective studies and studies on additional populations are needed to establish whether the use of this ratio in clinical practice, accompanied by the current OGTT at 24-28 gestational weeks, could reduce GDM, costs, or adverse pregnancy outcomes. Second, data on socioeconomic status, diet, and physical activities, which have been reported to be risk factors for GDM, were not included in this retrospective study [32].

## 5. Conclusions

In conclusion, this study shows that the machine learning algorithm does not outperform conventional logistic regressions. A prediction model with cutoff points was developed and provides values in risk assessment for detecting GDM.

## Figures and Tables

**Table 1:** Baseline characteristics.

Characteristic	Development set (*N* = 4900)			Validation set (*N* = 2100)		
	GDM (*N* = 2181)	Control (*N* = 2719)	*P* value	GDM (*N* = 1001)	Control (*N* = 1099)	*P* value
*Maternal characteristics*						
Maternal age (years)						
<20	1 (0.44%)	7 (0.26%)	<0.001	1 (0.11%)	0	<0.001
20-34.9	2051 (90.95%)	2522 (95.35%)		856 (92.34)	1105 (94.20%)	
35-40	159 (7.05%)	88 (3.33%)		51 (5.50%)	50 (4.26%)	
≥40	9 (0.40%)	3 (0.11%)		5 (0.54%)	1 (0.09%)	
BMI (kg/m^2^)						
<25	1290 (57.2%)	1920 (72.6%)	<0.001	559 (60.30%)	853 (72.7%)	<0.001
25-29.9	450 (20.0%)	269 (10.2%)		161 (17.40%)	110 (9.4%)	
>30	80 (3.5%)	26 (1.0%)		29 (3.10%)	7 (0.6%)	
Education status						
<College	740 (33.9%)	957 (35.2%)	0.41	372 (37.2%)	383 (34.8%)	0.28
College	1142 (52.4%)	1363 (50.1%)	0.08	491 (49.1%)	539 (49.0%)	0.97
>College	209 (9.6%)	299 (11.0%)	0.12	99 (9.9%)	132 (12.0%)	0.12
Smoking	30 (1.4%)	31 (1.1%)	0.44	14 (1.4%)	15 (1.4%)	0.85
Nulliparous	1840 (81.60%)	2194 (82.95%)	0.44	763 (82.31%)	973 (82.95)	0.67
Prior macrosomia	22 (1.0%)	15 (0.57%)	0.10	10 (1.08%)	3 (0.26%)	0.02
Prior preterm delivery	22 (1.0%)	17 (0.64%)	0.18	7 (0.76%)	10 (0.85%)	0.80
Prior GDM	20 (0.89%)	0	<0.001	12 (1.30%)	0	<0.001
Family history of diabetes	21 (0.93%)	9 (0.34%)	0.008	14 (1.51%)	3 (0.36%)	0.001
*Biochemical data*						
3-Triglyceride	1.67 ± 0.79	1.44 ± 0.59	<0.001	1.67 ± 0.85	1.40 ± 0.55	<0.001
Uric acid	213.30 ± 45.25	202.71 ± 39.88	<0.001	213.19 ± 44.93	203.24 ± 41.29	<0.001
Glycosylated hemoglobin	5.21 ± 0.43	5.03 ± 0.38	<0.001	5.19 ± 0.42	5.01 ± 0.35	<0.001
Alkaline phosphatase	67.85 ± 36.53	66.94 ± 35.94	0.008	68.06 ± 37.75	67.32 ± 36.85	0.07
Total cholesterol	4.71 ± 0.77	4.60 ± 0.76	<0.001	4.70 ± 0.86	4.65 ± 0.77	0.18
Lactic dehydrogenase	152.92 ± 37.80	153.00 ± 34.67	0.18	153.19 ± 34.77	152.38 ± 33.85	0.18
Fasting blood glucose	4.59 ± 0.67	4.34 ± 0.46	<0.001	4.59 ± 0.74	4.32 ± 0.40	<0.001
AFP concentration	42.82 ± 17.66	44.53 ± 17.32	<0.001	42.56 ± 16.19	44.72 ± 17.55	0.001
Fibrinogen	3.77 ± 0.64	3.63 ± 0.59	<0.001	3.72 ± 0.61	3.62 ± 0.62	<0.001
High-density lipoprotein	1.08 ± 0.22	1.05 ± 0.21	<0.001	1.08 ± 0.22	1.06 ± 0.22	0.04

Data are the *n* (%) or mean ± SD. *P* values indicate differences between groups calculated using the two-sample Wilcoxon rank-sum (Mann-Whitney) test for continuous variables and the Pearson *χ*^2^ test or ANOVA for categorical variables, with trend tests if appropriate. The “missing” category was not included in statistical tests. For characteristics that had no “missing” category, the data were 100% complete. Maternal age was defined as age at recruitment into the study. Maternal BMI was recorded at middle pregnancy when Down's syndrome screening was performed.

**Table 2:** Performance of the cutoff points of 0.3 and 0.7 for the GBDT model in predicting GDM.

Cutoff points	Development cohort Percent (95% CI)	Validation cohort Percent (95% CI)
0.3		
Negative predictive value	82.40% (79.90%-84.70%)	74.10% (69.50%-78.20%)
Positive predictive value	51.3% (49.80%-52.90%)	52.60% (50.20%-54.90%)
Sensitivity	92% (90.80%-93.10%)	90% (88.00%-91.70%)
Specificity	30% (28.30%-31.80%)	26% (23.50%-28.70%)
Positive likelihood ratio	1.31 (1.29-1.34)	1.22 (1.18-1.26)
Negative likelihood ratio	0.27 (0.11-0.42)	0.39 (0.17-0.60)
0.7		
Negative predictive value	59.30% (57.80%-60.70%)	56.1% (53.90%-58.30%)
Positive predictive value	86.60% (82.90%-89.60%)	93.2% (88.20%-96.10%)
Sensitivity	16% (14.50%-17.60%)	15% (12.90%-17.30%)
Specificity	98% (97.40%-98.50%)	99% (98.20%-99.40%)
Positive likelihood ratio	8 (7.72-8.28)	15 (14.38-15.61)
Negative likelihood ratio	0.86 (0.84-0.88)	0.86 (0.83-0.89)

**Table 3:** Performance of the cutoff points of 0.3 and 0.7 for the GBDT model in predicting adverse pregnancy outcomes.

Cutoff points	Development cohort Percent (95% CI)	Validation cohort Percent (95% CI)
0.3		
Negative predictive value	100% (92.7%-100%)	52.4% (32.4%-71.7%)
Positive predictive value	50.1% (48.7%-51.5%)	49.9% (47.8%-52.1%)
Sensitivity	100% (99.8%-100%)	99.0% (98.3%-99.5%)
Specificity	2% (1.5%-2.6%)	1% (0.6%-1.9%)
Positive likelihood ratio	1.02% (1.01%-1.03%)	1% (0.99%-1.01%)
Negative likelihood ratio	0 (0-nan)	1% (0.15%-1.85%)
0.7		
Negative predictive value	51.4% (50.0%-52.8%)	50.9% (48.7%-53.0%)
Positive predictive value	83.0% (76.1%-88.2%)	79.2% (66.5%-88.0%)
Sensitivity	5% (4.2%-6.0%)	4% (3.0%-5.4%)
Specificity	99% (98.5%-99.3%)	99% (98.1%-99.4%)
Positive likelihood ratio	5 (4.57-5.43)	4 (3.33-4.67)
Negative likelihood ratio	0.96 (0.95-0.97)	0.97 (0.96-0.98)

## Data Availability

The dataset generated and/or analyzed in this study is not publicly available, owing to currently ongoing research studies, but the data are available from the corresponding author on reasonable request.

## Supplementary Materials

Supplementary Materials Figure S1: discrimination and calibration of adverse pregnancy outcomes. Figure S2: distribution of predictive values for adverse pregnancy outcomes in the development and validation cohorts.
