# Supplementary material for: Comparison of Machine Learning Methods and Conventional Logistic Regressions for Predicting Gestational Diabetes Using Routine Clinical Data: A Retrospective Cohort Study
Source: J Diabetes Res. 2020 Jun 12;2020:4168340. doi: 10.1155/2020/4168340 (PMC7306091; doi:10.1155/2020/4168340)
Supplement: Supplementary Materials — Figure S1: discrimination and calibration of adverse pregnancy outcomes. Figure S2: distribution of predictive values for adverse pregnancy outcomes in the development and validation cohorts. [file 4168340.f1.pdf]

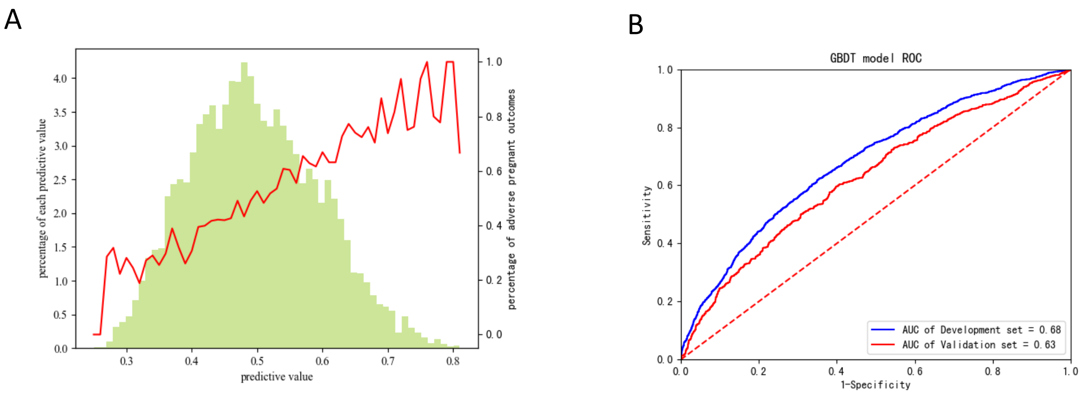

Figure S1. Discrimination and calibration of adverse pregnancy outcomes.

A. Discrimination is presented as the AUC.

B. The calibration curve indicates the association between predictive and observed predictive value for adverse pregnancy outcomes.

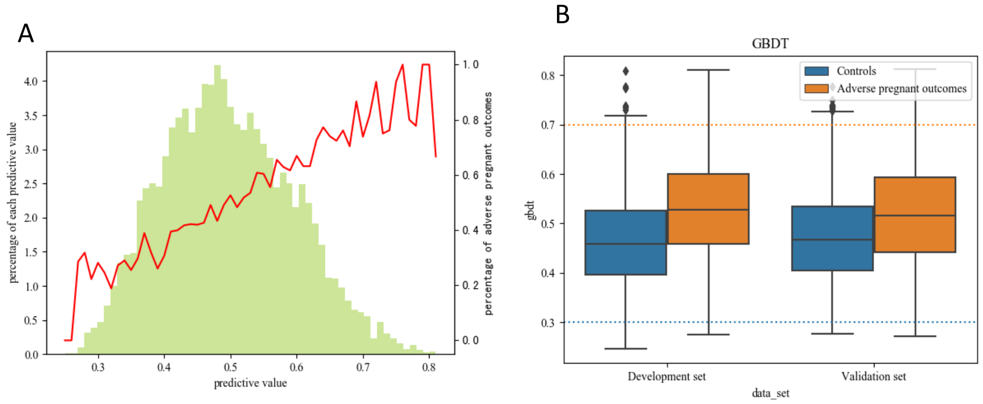

Figure S2. Distribution of predictive values for adverse pregnancy outcomes in the development and validation cohorts.

A. Distribution of predictive values and its relationship with the observed adverse pregnancy outcomes in the combined cohort (development and validation cohorts).

B. Distribution of predictive values in the development and validation cohorts. Data are presented as medians and interquartile ranges.
